# Daily Life Upper Limb Activity for Patients with Match and Mismatch between Observed Function and Perceived Activity in the Chronic Phase Post Stroke

**DOI:** 10.3390/s21175917

**Published:** 2021-09-02

**Authors:** Bea Essers, Marjan Coremans, Janne Veerbeek, Andreas Luft, Geert Verheyden

**Affiliations:** 1 Department of Rehabilitation Sciences, KU Leuven, 3001 Leuven, Belgium; marjan.coremans@kuleuven.be (M.C.); geert.verheyden@kuleuven.be (G.V.); 2 Luzerner Kantonsspital, Neurocenter, 6000 Lucerne, Switzerland; janne.veerbeek@luks.ch; 3 Division of Vascular Neurology and Neurorehabilitation, Department of Neurology and Clinical Neuroscience Center, University of Zurich and University Hospital Zurich, 8091 Zurich, Switzerland; andreas.luft@usz.ch; 4 Cereneo, Center for Neurology and Rehabilitation, 6354 Vitznau, Switzerland

**Keywords:** stroke, upper limb sensor activity, patient-reported outcome measures

## Abstract

We investigated actual daily life upper limb (UL) activity in relation to observed UL motor function and perceived UL activity in chronic stroke in order to better understand and improve UL activity in daily life. In 60 patients, we collected (1) observed UL motor function (Fugl-Meyer Assessment (FMA-UE)), (2) perceived UL activity (hand subscale of the Stroke Impact Scale (SIS-Hand)), and (3) daily life UL activity (bilateral wrist-worn accelerometers for 72 h) data. Data were compared between two groups of interest, namely (1) good observed (FMA-UE >50) function and good perceived (SIS-Hand >75) activity (good match, *n* = 16) and (2) good observed function but low perceived (SIS-Hand ≤75) activity (mismatch, *n* = 15) with Mann–Whitney *U* analysis. The mismatch group only differed from the good match group in perceived UL activity (median (Q1–Q3) = 50 (30–70) versus 93 (85–100); *p* < 0.001). Despite similar observed UL motor function and other clinical characteristics, the affected UL in the mismatch group was less active in daily life compared to the good match group (*p* = 0.013), and the contribution of the affected UL compared to the unaffected UL for each second of activity (magnitude ratio) was lower (*p* = 0.022). We conclude that people with chronic stroke with low perceived UL activity indeed tend to use their affected UL less in daily life despite good observed UL motor function.

## 1. Introduction

One of the most important outcomes for stroke survivors, carers, and clinicians is being able to perform everyday tasks, which may be hampered by upper limb (UL) problems [1]. Only a limited number of stroke survivors at six months post stroke are able to fully re-engage the affected UL in daily activities [2]. In order to re-engage the UL in daily life post stroke, a certain level of UL motor function is needed. Cross-sectional studies in the chronic phase post stroke have shown that patients with low to moderate UL motor function use their paretic UL less in daily life than those with good UL function [3,4,5]. A longitudinal study in the first six months post stroke further shows that affected UL use in daily life increases more in patients with excellent UL function recovery than in those with mild or moderate recovery [4]. It thus seems that one’s UL motor function needs to reach a threshold in order to use the UL in daily life [5].

Good UL motor function, however, does not translate into a similar daily life UL use per se. In the subacute phase, a wide variation in daily UL use can be seen in patients with good UL function [6,7], and improved UL function does not directly result in increased daily UL use [8]. These discrepancies seem to persist into the chronic phase post stroke [9,10,11], which suggests that factors in addition to UL motor function influence daily life UL use. Factors such as motivation, health behaviors, environmental supports, comorbidities, psychosocial support, and neglect have been reported, but authors have not further investigated their relationship with UL use in daily life [11,12]. In one cross-sectional study, the authors examined potentially modifying factors of affected UL activity in community-dwelling adults with chronic stroke and found that, next to severity of motor dysfunction, only dependence in activities of daily living was associated with affected UL activity [13].

A factor that is often overlooked but may nevertheless play an important role in the use of one’s UL motor function is the perceived UL activity. How a person perceives his or her ability to use the UL in daily life can be measured with patient-reported outcome measures and is strongly correlated with the patient’s observed UL motor function as assessed by a clinician [14]. However, despite this strong correlation, the observed UL function and perceived UL activity are not always congruent. In a cross-sectional study, patient-reported outcomes revealed deficits in daily life UL activity in a majority of patients with stroke who did not seem to have deficits based on observation-based assessments [15]. Further, we recently investigated the correspondence between perceived UL activity and observed UL function and found that in two third of the patients at 12 months post stroke, observed and perceived assessments correspond well, namely people with a low observed function have a similar low perceived activity (low match group), whereas people with good observed function report good perceived activity (good match group). However, next to these two match groups, one in three patients have good observed function but low perceived UL activity, that is, the mismatch group [16]. 

A mismatched perception of poor UL motor function despite actually having good UL function might result in reduced UL activity. We thus wanted to investigate if there is a difference in daily life UL activity between patients with good observed UL motor function and low perceived UL activity compared to those with corresponding low or good abilities. Therefore, we cross-sectionally investigated individuals from the low match, good match, and mismatch groups six months post stroke with observation-based assessments, patient-reported outcomes, and assessments of daily life UL activity. We hypothesized that people in the mismatch group will show higher daily life UL activity compared to those in the low match group but reduced daily life UL activity compared to those in the good match group. For this mismatch group to make full use of their capabilities in daily life, a specific therapeutic approach would be required.

## 2. Materials and Methods

### 2.1. Participants

Adults with chronic stroke participated in this cross-sectional cohort study. They were recruited between October 2020 and May 2021 from the discharge records of the University Hospitals Leuven rehabilitation center Pellenberg and our own database of participants with stroke from previous studies. To further widen our recruitment pool, we encouraged private physiotherapy practices over e-mail to inform potential participants about the study. Inclusion criteria were (1) first-ever, unilateral, supratentorial stroke as defined by the World Health Organization (WHO) [17], (2) minimum six months after stroke, (3) living in the community regardless of their level of disability, and (4) ≥18 years old. Patients were excluded if they had (1) a musculoskeletal and/or other neurological disorder, such as head injury or Parkinson’s disease, that interferes with the protocol and (2) severe communication or cognitive deficits. This study was approved by the Ethics Committee Research (EC Research) of University Hospitals Leuven (UZ Leuven) and was prospectively registered at https://clinicaltrials.gov, accessed on 26 July 2021, identifier NCT04430153. All participants provided written informed consent in accordance with the Declaration of Helsinki and were compensated for their time with a bookstore voucher.

We recruited participants with (1) low observed and low perceived functioning (low match group), (2) good observed and good perceived functioning (good match group), and (3) good observed but low perceived functioning (mismatch group). Given the exploratory nature of this study, a sample size calculation was not possible. Instead, sample size was guided by the 12-month result of our recent longitudinal study showing an equal distribution of about one third of participants in each of the three groups [16]. We thus planned a recruitment of 20 participants for each group, with a total sample size of 60.

### 2.2. Definitions

Throughout the manuscript, several terms have been frequently used to describe the upper limb functioning. In Table 1, we define those key terms.

### 2.3. Procedure

Participants completed a 1.5 to 2 h visit at home or in the lab depending on their preference. First, they provided demographic and health information, including date of birth, gender, time post stroke, lateralization of symptoms, and pre-stroke hand dominance. Then, the observed UL motor function was assessed with the reliable and valid upper extremity subscale of the Fugl-Meyer Assessment (FMA-UE) [21] and the perceived UL activity with the hand subscale of the Stroke Impact Scale version 3.0 (SIS-Hand) [22]. To recruit participants from the different (mis)match groups, we stratified them based on our previously defined cut-off scores for the observed UL motor function and perceived UL activity [23]. The cut-off score was 31/66 on the FMA-UE to discriminate between low (≤31) and good (>31) observed UL motor function [24] and 61/100 on the SIS-Hand to discriminate between low (≤61) and good (>61) perceived UL activity [25].

However, a recently published paper investigating UL use in people in the early phase post stroke showed that people with FMA-UE scores higher than 50 have significantly higher UL use compared to those with a score between 23 and 50 [7]. This cut-off of 50/66 was also used in previous studies to distinguish moderate and mild impairments [26,27,28]. Further, in a longitudinal study investigating the SIS-Hand in 101 chronic stroke patients, the average score was 70.85 at six months and 77.53 at 12 months [29]. Thus, it might be that our previously defined cut-off scores would be too low to distinguish patients with good UL functioning. Therefore, we decided to compare our two groups of interest based on the new cut-off values, namely the mismatch group (FMA-UE >50, SIS-Hand ≤75) and the good match group (FMA-UE >50, SIS-Hand >75). Between-group comparisons based on the previously defined cut-off values used to include participants in this cross-sectional trial are given in the Appendix A.

After clinical examination, accelerometers were placed on both wrists proximal to the ulnar styloid. Participants were instructed to wear the accelerometers for the subsequent 72 h [30] (including sleep [31]), while they went about their normal, daily routines. They were permitted to remove the devices when swimming or bathing. After the wearing period, accelerometers were picked up by the researcher along with the wearing log. In this log, the patients were asked to note when they removed the sensors and for what reason and when they had physical or occupational therapy.

### 2.4. Accelerometry

Wrist-worn accelerometry was used to measure UL activity as it has established reliability and validity in adults with stroke [30,32,33]. Two triaxial accelerometers (wGT3X-BT Activity Monitor, Actigraph Inc., Pensacola, FL, USA) were placed on each wrist proximal to the styloid process of the ulna. Acceleration was recorded along three axes at a selected frequency of 30 Hz and stored on the accelerometers in a raw, nonfiltered format in the units of gravity (G’s). These raw data were downloaded to the computer and postprocessed using the ActiLife 6 software (Actigraph Inc., Pensacola, FL, USA). This software band pass filtered the data between frequencies of 0.25 and 2.5 Hz, accumulated data into 1 s epochs, and converted them into activity counts (0.001664 g/count) [34,35]. For each second of data, activity counts across the three axes were combined into a single value, called a vector magnitude ($\sqrt{x^{2}+y^{2}+z²}$).

Before data analysis, we visually inspected the data in ActiLife to ensure that the accelerometers were worn for the planned time period and that the data matched the wearing log. Based on the wearing logs, we manually deleted data collected during physiotherapy or occupational therapy and during nonwear periods [36]. Afterwards, we created accelerometry metrics and graphical representations as described in detail in “a method for quantifying UL activity in daily life using accelerometers” [37]. In short, we calculated (1) hours of activity for each limb by summing all seconds recorded when the activity count was nonzero and converting them to hours; (2) the activity ratio by dividing hours of activity of the affected by the unaffected limb; (3) magnitude ratio by taking the natural log of the vector magnitude of the affected limb divided by the vector magnitude of the unaffected limb, whereby values greater than and less than −7 were replaced by 7 and −7, respectively, to categorize single limb movement; and (4) bilateral magnitude by summing the vector magnitude from the two limbs. The activity ratio and magnitude ratio give insight into the contribution to the activity of one limb versus the other, whereas the bilateral magnitude indicates the intensity of the movement. We then constructed density plots for the three groups by combining 24-h data of each participant with the magnitude ratio on the x-axis and the bilateral magnitude on the y-axis. Each second of data is shown on the plot as a bivariate histogram with the frequency represented in color.

Last, we calculated two extra accelerometry metrics based on a previous study quantifying real world UL activity in chronic stroke [9], namely (1) unilateral paretic and nonparetic hours of UL activity by summing seconds when only one UL was active and (2) bilateral UL activity by summing seconds when both ULs were active. For the three “hours of activity” variables, we averaged the three days of accelerometry data to 24 h to obtain insight into the daily activity. All accelerometry outcomes were calculated using custom scripts created in Matlab R2020a (Mathworks, Nattick, IL, USA) software.

### 2.5. Statistical Analysis

Normality was checked for all variables with the Shapiro–Wilk test. Mean and standard deviations (SD) were calculated for normally distributed variables, medians with first quartile (Q1) and third quartile (Q3) for non-normally distributed variables, and frequencies with percentages for counts. Accelerometry-derived variables were compared between groups through parametric (one-way ANOVA and subsequent independent *t*-test) or nonparametric (Kruskal–Wallis and subsequent Mann–Whitney *U* test) analysis. Data were analyzed with IBM SPSS Statistics for Windows, version 27.0 (IBM Corp, Armonk, NY, USA) with the level of two-tailed statistical significance set at *p* < 0.05. The relative magnitude of the differences between groups was calculated using mean or median differences with their 95% confidence interval (CI). In order to reduce differences between the mismatch group and the good match group due to our inability to randomly assign the participants to different groups, we searched the literature for potential confounders. Variables found in the literature that are believed to be predictive of exposure status (mismatch versus good match, i.e., low versus high perceived UL activity) or the outcome (actual UL activity) in the chronic stroke population are dependence in ADL [13], gender [38], and concordance (dominant side = affected) [11,13]. Increased dependence in ADL was associated with decreased affected UL activity [13], being female was associated with lower increases in UL activity outcomes after rehabilitation [38], and UL activity values were lower in participants whose nondominant UL was affected than in participants whose dominant UL was affected [11,13]. Another factor that is associated with reduced levels of physical activity in adults with stroke is depression [39]. We checked if these variables were related with our dependent variable (UL activity outcomes) and included them as confounder in an analysis of covariance (ANCOVA). This study conformed to the STROBE guidelines [40] and has reported the required information accordingly.

## 3. Results

### 3.1. Participant Characteristics

**Figure 1:**
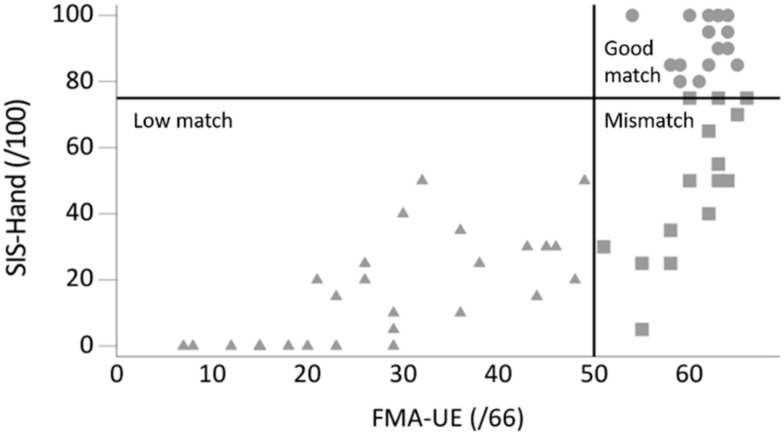
Graph plotted with perceived upper limb activity (SIS-Hand: hand domain of the Stroke Impact Scale) against observed upper limb function (FMA-UE: Fugl-Meyer Assessment upper extremity) score showing three groups: low match (*n* = 29; FMA-UE ≤50, SIS-Hand ≤75), mismatch (*n* = 15; FMA-UE >50, SIS-Hand ≤75), and good match (*n* = 16; FMA-UE >50, SIS-Hand >75).

Sixty-two people with stroke were recruited. One participant was excluded due to bilateral deficits and another participant had no data recorded for the affected UL due to technical problems with the sensor. From the remaining 60 stroke participants, 29 were in the low match group, 15 in the mismatch group, and 16 in the good match group (Figure 1). Demographic information and stroke-specific characteristics are displayed in Table 2. The mean age of our sample was 62 years (Q1–Q3 = 50–67), 62% (*n* = 37) were male, 47% (*n* = 28) had a right-sided hemiparesis, and 62% (*n* = 37) had suffered an ischemic stroke. The median time since stroke onset was 977 days (Q1–Q3 = 577–1618) and all patients were community-dwelling adults.

None of the demographic or stroke-related characteristics were different between the mismatch group and the good match group, except for the higher depression and anxiety values and a lower number of people with the dominant hand affected in the mismatch group (Table 2).

For the UL motor function, observed values as well as the perceived UL activity were higher in the mismatch group compared to the low match group (*p* < 0.001). The observed UL motor function, on the other hand, was not different between the mismatch group and the good match group (FMA-UE median = 62; Q1–Q3 = 58–63 versus median = 62; Q1–Q3 = 60–64)). However, the perceived UL activity was higher in the good match group (median = 93; Q1–Q3 = 85–100) compared to the mismatch group (median = 50; Q1–Q3 = 30–70, *p* < 0.001) (Table 3).

### 3.2. Ul Activity across The Three Groups

Participants wore the accelerometers for the designated wearing period (median = 72; Q1–Q3 = 72–72 h). Comparing UL activity across the three groups, all variables were significantly different, except for duration of unaffected UL activity (Table 3). Comparing the mismatch group to the low match group, post-hoc tests revealed that all UL activity outcomes were significantly better in the mismatch group except for hours of unaffected UL activity and median bilateral magnitude. The last two were higher in the mismatch group compared to the low match group, but differences were not significant (*p* > 0.05).

**Figure 2:**
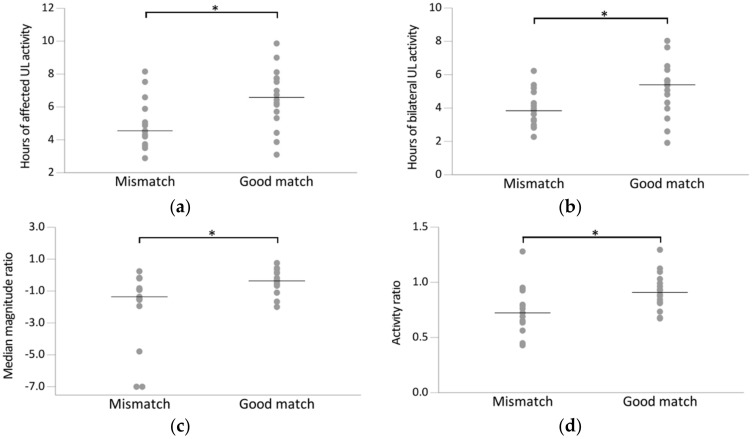
Scatterplot of accelerometry outcome variables for the mismatch group (*n* = 15; FMA-UE >50, SIS-Hand ≤75) and good match group (*n* = 16; FMA-UE >50, SIS-Hand >75). Every dot represents the raw value of a patient; raw mean (**a**–**c**) or median scores (**d**) indicated with horizontal bar. Horizontal brackets indicate significant differences between two groups (*p* < 0.05). (**a**) Hours of affected UL activity, (**b**) hours of bilateral UL activity, (**c**) median magnitude ratio, (**d**) activity ratio.

Comparing the mismatch to the good match group, the unaffected UL activity was relatively higher in the good match group (mean = 7.2, SD = 1.8) compared to the mismatch group (mean = 6.8, SD = 1.4), but this difference was not significant (*p* = 0.472). The affected UL, on the other hand, was more active in the good match group (mean = 6.6 h, SD = 1.8) compared to the mismatch group (mean = 5, SD = 1.5, *p* = 0.013) (Figure 2a). Moreover, during unilateral activities, the affected UL was less active in the mismatch group (mean = 1 h, SD = 0.7) compared to the good match group (mean = 1.4, SD = 0.6, *p* = 0.092), whereas the unaffected UL was more active during unilateral activities in the mismatch group (mean = 2.8 h, SD = 1.3) compared to the good match group (mean = 2, SD = 0.8, *p* = 0.05). Lastly, the number of hours in which both ULs were active was higher in the good match group (mean = 5.1 h, SD = 1.7) compared to the mismatch group (mean = 3.9, SD = 1.1, *p* = 0.025) (Figure 2b).

For the ratio variables, both the activity ratio and the median magnitude ratio were higher in the good match group compared to the mismatch group (*p* = 0.020 and *p* = 0.022, respectively) (Figure 2c–d), indicating that the increased activity of the unaffected UL relative to the affected UL was higher in the mismatch group compared to the good match group. Lastly, the intensity of UL activity was higher in the good match group compared to the mismatch group as indicated by the higher median bilateral magnitude value, although this was not significant (*p* = 0.063).

As none of the potential confounders defined in the Methods section was correlated with the dependent UL activity variables in our sample, which was an assumption made to perform ANCOVA, we did not perform a confounder analysis in the end.

**Figure 3:**
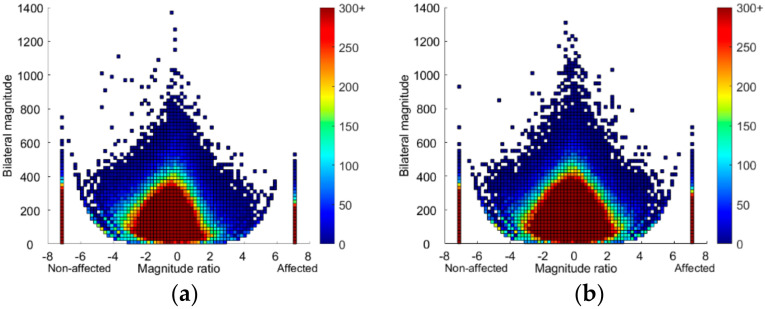
Density plots showing 24 h of upper limb activity in the (**a**) mismatch group (*n* = 15; Fugl-Meyer Assessment upper extremity FMA-UE >50, hand domain of the Stroke Impact Scale SIS-Hand ≤75) and (**b**) good match group (*n* = 16; FMA-UE >50, SIS-Hand >75) plotted on a second-by-second basis. The magnitude ratio (x-axis) indicates contribution of each limb to activity. The bilateral magnitude (y-axis) indicates the activity intensity. The color represents frequency, with brighter colors indicating greater frequencies. Bars at −7 and 7 show unilateral unaffected and affected UL activity, respectively.

Figure 3 shows density plots of 24-h UL activity for the mismatch group and the good match group, supporting the statistics in Table 3. The mismatch group (Figure 3a) showed a slightly more asymmetrical plot compared to the good match group (Figure 3b), indicating that both unilateral and simultaneous UL activity consisted more of unaffected UL activity. Next, the central peak in the mismatch plot was lower than in the good match plot, indicating that the bilateral UL tasks for the mismatch group were less intense compared to the good match group.

## 4. Discussion

This study investigated whether there is a difference in daily life UL activity between patients with good observed UL motor function and low perceived UL activity (mismatch group) compared to those with corresponding low or good abilities (low and good match group, respectively). As hypothesized, people in the mismatch group showed a higher daily life UL activity compared to those in the low match group but a reduced daily life UL activity compared to those in the good match group. The only variables that did not differ between groups was the amount of unaffected UL activity and the median bilateral magnitude, which is in line with previous studies in the subacute [7] and chronic phase [3,11] post stroke.

The overall lower accelerometry outcomes in the low match group compared to the mismatch group were not surprising given the large difference in observed as well as perceived UL motor function. The observed UL motor function in the mismatch group was 55% higher than that for participants in the low match group, in which participants had severe UL impairment (FMA-UE range 7–49). It might be that the affected UL motor function in the low match group did not reach the threshold in order for this limb to be used in daily life [5]. On top of that, the difference in perceived UL motor activity was 40% lower in the low match group compared to the mismatch group, which may have contributed to the difference in UL activity outcomes.

Our main interest though was the comparison between the mismatch group and the good match group and to examine whether there is a difference in UL activity between people who have a similar good UL motor function but different perceived UL activity.

**Figure A1:**
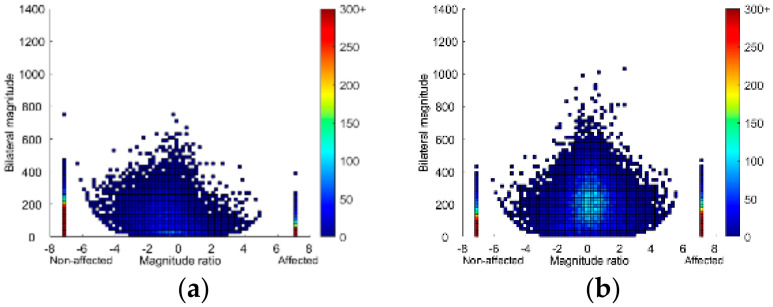
Density plots showing 24 h of upper limb activity for a single person in the (**a**) mismatch group (Fugl-Meyer Assessment upper extremity FMA-UE = 58, hand domain of the Stroke Impact Scale SIS-Hand = 25) and (**b**) good match group (FMA-UE = 59, SIS-Hand = 80), plotted on a second-by-second basis. The magnitude ratio (x-axis) indicates contribution of each limb to activity. The bilateral magnitude (y-axis) indicates the activity intensity. The color represents frequency, with brighter colors indicating greater frequencies. Bars at −7 and 7 show unilateral unaffected and affected UL activity, respectively.

The median perceived UL activity was 43% higher in the good match group compared to the mismatch group, but the observed UL motor function was similar (median FMA-UE 62 in both groups). Interestingly, we saw higher UL activity outcomes for the good match group compared to the mismatch group. This was supported by the density plots, that is, the mismatch plot was more asymmetrical, whereas the symmetrical plot from the good match group was similar to plots in healthy older population [9]. Compared to plots from individual participants in previous research, our plots had warmer colors, indicating higher frequency of UL activity. This can be explained by a difference in method, whereby we combined 24-h data for all participants in each group in order to compare plots at a group level rather than for each individual separately (Appendix A, Figure A1). Where values for the mismatch group were comparable to those previously reported in a chronic stroke sample, values for the good match group were higher [13]. In the previous study, however, participants over the entire range of UL motor abilities were included and not stratified, which prevented direct comparison to our participants with good observed UL motor function. On the other hand, compared to healthy older adults, the good match group was similar but the mismatch group had a much lower activity ratio and median magnitude ratio [31].

The lower UL activity in the mismatch group compared to the good match group might be due to a lower perceived UL activity. A lower perceived UL activity, or a lower belief in one’s motor ability, might be linked to a lower self-efficacy [41]. A lower self-efficacy, or confidence in UL activity, has been shown to affect paretic UL activity during early stroke rehabilitation, with higher self-efficacy related to greater paretic UL use [8,42]. Moreover, in the chronic phase post stroke, greater self-efficacy is associated with reduced nonuse of the paretic UL in daily life [43]. Increased perceived ability might thus promote behavioral change needed to encourage paretic UL activity in daily life [44]. The difference in UL activity might further be increased by the difference in depression and anxiety and independence in daily activities. However, these variables did not seem to be correlated in our sample with the UL activity outcomes. This indicates that, although depression and anxiety may be higher and the independence in daily activities lower in the mismatch group compared to the good match group, it does not explain the difference in UL activity values between the two groups.

The fact that the bilateral magnitude did not differ significantly between the mismatch group and the good match group whereas the magnitude ratio and activity ratio did may be explained by a difference in construct. Both the magnitude ratio as well as the activity ratio are called “symmetry measures” as they give an idea if the left and right ULs are equally contributing and active during daily activities [45]. While these symmetry values have a narrow range in the typical adult population, there is evidence of it being different in populations with neuromotor impairment [45] and between groups with different motor function [46]. The bilateral magnitude, on the other hand, indicates the intensity of the movement. It was previously shown that the bilateral magnitude was only significantly different between mild and severe groups in the acute phase post stroke [7]. It seems that once a certain amount of UL function is achieved (FMA-UE >50), the intensity of UL activity does not increase that much anymore. 

A strength of this study compared to previous research on the chronic phase post stroke is that we combined observation-based assessments, patient-reported outcomes, and assessments of daily life UL activity in a considerable group of patients with a wide range of UL motor functioning. A limitation of the study is inherent to the use of accelerometry as an assessment of daily life UL activity. On the one hand, accelerometers may overestimate UL movements, such as arm swing during ambulation and acceleration in a car. However, the ActiLife software band pass filters to isolate and exclude these movements [47], and ratios of affected-to-unaffected UL activity adequately control for walking [36]. On the other hand, wrist-worn accelerometry may miss small movements made with the hands and fingers that occur without moving more proximal segments [46]. However, many UL movements require coordinated multi-joint activity and will be captured using wrist-worn accelerometers [48]. 

Next, as in most studies, our sample of patients represents a subgroup within the chronic stroke population. However, we included patients in the full range of the SIS-Hand scale and nearly full range of the FMA-UE scale. Although we consecutively included patients with all combinations of SIS-Hand and FMA-UE scores until group completion, we did not meet participants with a low FMA-UE score and good SIS-Hand score. This is in contrast with other studies, in which a second mismatch group of patients with low observed and good perceived scores was described [49,50,51]. This may be explained by a difference in study design and timing post stroke. The two previous studies measured improvement over time, whereas we used a cross-sectional methodology [49,51]. Another study was performed in the early phase post stroke [50], in which people might have overestimated their possibilities more than the community-dwelling participants with chronic stroke in our study.

Finally, due to the observational design, this study could not demonstrate cause–effect relationship between perceived UL activity and daily UL activity. Nonetheless, this study confirms the finding that UL motor activity in daily life is not a direct reflection of UL motor ability as previously demonstrated [9,10,11]. It further points out the importance of perceived UL activity among patients with good observed UL function, that is, people with good observed but low perceived UL functioning could potentially achieve a higher level of daily life UL activity by focusing on improving perceived UL activity. Therapy should thus try to incorporate principles that can improve perceived UL activity in order to fully re-engage the affected UL in daily life post stroke.

## 5. Conclusions

The present study contributes to better understanding of UL activity in the chronic phase post stroke for patients with either a match or a mismatch between observed and perceived UL abilities, which is essential for optimizing UL activity in daily life after stroke. The study confirms that a certain amount of UL motor function is needed for the UL to be active in daily life in the chronic phase post stroke. However, despite similar good observed UL function, greater UL activity outcomes in the group with good perceived UL activity compared to the group with low perceived activity indicate that good observed UL function does not always translate to good UL activity in daily life. It furthermore shows that the patient’s perception of ability to use the arm in daily life matches daily life arm activity as assessed by movement sensors. The lower UL activity outcomes in the mismatch group could be due to a mismatch between actually having good UL function and perceiving the UL function as poor, that is, good perceived UL activity might be a mediating factor in using the good observed UL function in daily life. This study has several clinical implications. First, it underlines the importance of assessing UL activity in daily life using patient-reported outcome measures and, if available, accelerometers to add objective information instead of simply relying on observed UL function in private practice. Second, assessing perceived UL motor activity may be important to derive a complete understanding of daily life UL activity, especially among patients with good observed UL function. Third, in patients with good observed UL function but low perceived UL activity, efforts to increase perceived UL activity and independence in daily activities should be investigated and incorporated for use in clinical practice. These efforts might include behavioral methods, such as contracting, self-monitoring, problem solving, and home skill assignments, as they have been suggested to increase patients’ perceived UL activity [52,53]. Further, it may be important to regularly give feedback as this can improve self-efficacy [54], which is associated with increased perceived health status, lower depression, better activities in daily living, and reduced nonuse [43,55], which are all aspects worthy of attention in the mismatch group.

## Figures and Tables

**Table 1:** Definitions for key terms.

Upper limb functioning	An umbrella term for all upper limb functions, activities, and participation [18].
Observed upper limb motor function	The physiological function of the upper limb as observed and scored by the therapist [18].
Perceived upper limb activity	The level of upper limb activity subjectively experienced by a person at a given moment in his/her current environment [19].
Daily life upper limb activity	Real-world upper limb movement that is measured by accelerometry [20].

**Table 2:** Demographic and stroke-related characteristics presented as median (Q1–Q3) or number (%).

	Low Match (*n* = 29)	Mismatch (*n* = 15)	Good Match (*n* = 16)
Age at stroke onset (years)	63 (52–67)	56 (45–71)	63 (54–68)
Gender (male)	19 (66)	7 (47)	11 (67)
Dominant hand affected	13 (45)	4 (27)	10 (63)
Hand dominance (right)	25 (86)	13 (87)	15 (94)
Accommodation			
Living alone	15 (52)	5 (33)	9 (56)
Living not alone	14 (48)	10 (66)	7 (44)
Level of education			
Lower secondary education	4 (14)	1 (7)	2 (13)
Higher secondary education	14 (48)	5 (33)	6 (38)
Higher tertiary education	8 (28)	8 (53)	4 (25)
University degree	2 (7)	1 (7)	3 (19)
PhD degree	1 (3)	0 (0)	1 (6)
Employment status, working	4 (14)	4 (27)	6 (38)
Marital status			
Married	18 (62)	11 (73)	8 (50)
Divorced	3 (10)	1 (7)	3 (19)
Living together	2 (7)	0 (0)	2 (13)
Unmarried	1 (3)	2 (13)	2 (13)
Single	4 (14)	1 (7)	1 (6)
Widow(er)	1 (3)	0 (0)	0 (0)
Days since stroke onset	991 (673–1920)	846 (429–1489)	975 (545–1345)
Lateralization (left hemisphere)	14 (48)	5 (33)	9 (56)
Stroke etiology (ischemia)	16 (55)	11 (73)	10 (63)
NIHSS sensation			
No sensory loss	11 (38)	8 (53)	13 (81)
Mild to moderate sensory loss	17 (59)	5 (33)	3 (19)
Severe to total sensory loss	1 (3)	2 (13)	0 (0)
Disability (mRS)			
0: No symptoms at all	0 (0)	0 (0)	2 (13)
1: No significant disability despite symptoms	0 (0)	5 (33)	8 (50)
2: Slight disability	12 (41)	7 (47)	6 (38)
3: Moderate disability	13 (45)	3 (20)	0 (0)
4: moderately severe disability	4 (14)	0 (0)	0 (0)
Dependence in ADL (Barthel Index/100)	85 (70–95)	100 (95–100)	100 (100–100)
Cognitive function (MoCA/30)	23 (19–27)	26 (24–27)	27 (24–28)
Depression and anxiety (HADS/42)	11 (6–15)	12 (8–20)	8 (4–12)
Anxiety (HADS anxiety/21)	5 (3–7)	6 (5–9)	4 (2–7)
Depression (HADS depression/21)	6 (2–9)	6 (3–10)	4 (1–6)
Neglect (SCT <44/54)	3 (10)	1 (7)	0 (0)

ADL: activities of daily living; FMA-UE: Fugl-Meyer Assessment upper extremity; good match: FMA-UE >50, SIS-Hand >75; HADS: Hospital Anxiety and Depression Scale; low match: FMA-UE ≤50, SIS-Hand ≤75; mismatch: FMA-UE >50, SIS-Hand ≤75; mRS: modified Rankin Scale; MoCA: Montreal Cognitive Assessment; NIHSS: National Institutes of Health Stroke Scale; Q1: 1st quartile; Q3: 3rd quartile; SCT: Star Cancellation Test; SIS-Hand: hand domain of the Stroke Impact Scale.

**Table 3:** Upper limb functioning and accelerometry outcomes over 24 h across the three groups presented as mean (SD) or median (Q1–Q3).

	Low Match (*n* = 29)	Mismatch (*n* = 15)	Good Match (*n* = 16)	*p* Value across Three Groups	*p* Value Low Match vs. Mismatch	Mean/Median Difference (95% CI)	*p* Value Good Match vs. Mismatch	Mean/Median Difference (95% CI)
Observed UL motor function (FMA-UE/66) ^b^	26 (15–36)	62 (58–63)	62 (60–64)	<0.001 *	<0.001 **	−36 (−40; −26)	0.564	1 (−1; 4)
Perceived UL activity (SIS-Hand/100) ^b^	10 (0–25)	50 (30–70)	93 (85–100)	<0.001 *	<0.001 **	−40 (−50; −25)	<0.001 **	45 (30; 55)
Hours of affected UL activity during 24 h ^a^	2.9 (1.7)	5 (1.5)	6.6 (1.8)	<0.001 *	<0.001 **	−2 (−3.1; −1)	0.013 **	1.6 (0.4; 2.8)
Hours of unaffected UL activity during 24 h ^a^	6.6 (2.3)	6.8 (1.4)	7.2 (1.8)	0.628	0.792	−0.2 (−1.5; 1.2)	0.472	0.4 (−0.8; 1.6)
Hours of unilateral affected UL activity during 24 h ^a^	0.4 (0.3)	1 (0.7)	1.4 (0.6)	<0.001 *	0.004 **	−0.6 (−1; −0.2)	0.092	0.4 (−0.1; 0.9)
Hours of unilateral unaffected UL activity during 24 h ^a^	4 (1.2)	2.8 (1.3)	2 (0.8)	<0.001 *	0.003 **	1.2 (0.4; 2)	0.050	−0.8 (−1.5; 0)
Hours of bilateral UL activity during 24 h ^a^	2.6 (1.6)	3.9 (1.1)	5.1 (1.7)	<0.001 *	0.004 **	−1.4 (−2.3; −0.5)	0.025 *	1.2 (0.2; 2.2)
Activity ratio ^a^	0.42 (0.15)	0.75 (0.22)	0.92 (0.17)	<0.001 *	<0.001 **	−0.3 (−0.4; −0.2)	0.020 **	0.17 (0.03; 0.31)
Median bilateral magnitude ^b^	81.1 (58.7−96.4)	93.1 (74−101.1)	105.3 (87.5−140.3)	0.002 *	0.069	−12 (−32.3; 1)	0.063	13.3 (−0.7; 39.5)
Median magnitude ratio ^b^	−7 (−7–−7)	−1.36 (−1.94–−0.21)	−0.36 (−0.64–0.16)	<0.001 *	<0.001 **	−5.6 (−6.1; −3.1)	0.022 **	1 (0.3; 1.7)

FMA-UE: Fugl-Meyer Assessment upper extremity; good match: FMA-UE > 50, SIS-Hand > 75; low match: FMA-UE ≤ 50, SIS-Hand ≤ 75; mismatch: FMA-UE > 50, SIS-Hand ≤ 75; Q1: 1st quartile; Q3: 3rd quartile; SIS-Hand: hand domain of the Stroke Impact Scale; 95% CI: 95% confidence interval. * *p* < 0.05, ** *p* < 0.025 (Bonferroni correction for multiple testing). ^a^ One-way ANOVA; post-hoc: independent *t*-test. ^b^ Kruskal–Wallis H; post-hoc: Mann–Whitney *U* test.

## Data Availability

The data analyzed during the current study are available from the corresponding author, Bea Essers, upon reasonable request.

## Appendix A

**Table A1:** Upper limb functioning and accelerometry outcomes over 24 h across the three groups presented as mean (SD) or median (Q1–Q3) based on the old cut-off values.

	Low Match (*n* = 19)	Mismatch (*n* = 20)	Good Match (*n* = 21)	*p* Value across Three Groups	*p* Value Low Match vs. Mismatch	Mean/Median Difference (95% CI)	*p* Value Good Match vs. Mismatch	Mean/Median Difference (95% CI)
Observed UL motor function (FMA-UE/66) ^b^	20 (15–26)	50 (43–60)	62 (60–64)	<0.001 *	<0.001 **	31 (24; 37)	<0.001 **	11 (5; 17)
Perceived UL activity (SIS-Hand/100) ^b^	0 (0–15)	30 (25–50)	85 (78–100)	<0.001 *	<0.001 **	30 (20; 35)	<0.001 **	55 (45; 65)
Hours of affected UL activity ^a^	2.7 (1.6)	4.3 (2)	6.1 (1.8)	<0.001 *	0.017 **	1.6 (0.3; 3)	0.006 **	1.8 (0.5; 3.2)
Hours of unaffected UL activity ^a^	6.6 (2.4)	6.8 (2)	7 (1.7)	0.794	0.943	0.2 (−1.4; 1.8)	0.933	0.2 (–1.3; 1.7)
Hours of unilateral affected UL activity ^a^	0.4 (0.3)	0.7 (0.7)	1.3 (0.6)	<0.001 *	0.104	0.4 (−0.1; 0.8)	0.008 **	0.6 (0.1;1)
Hours of unilateral unaffected UL activity ^a^	4.3 (1.2)	3.2 (1.3)	2.2 (0.8)	<0.001 *	0.015 **	1 (0.2; 1.9)	0.012 **	1 (0.2; 1.9)
Hours of bilateral UL activity ^a^	2.3 (1.5)	3.6 (1.6)	4.8 (1.6)	<0.001 *	0.035	1.3 (0.1; 2.5)	0.031	1.3 (0.1; 2.4)
Activity ratio ^a^	0.38 (0.14)	0.63 (0.25)	0.87 (0.17)	<0.001 *	<0.001 **	0.25 (0.10; 0.40)	<0.001 **	0.24 (0.10; 0.39)
Median bilateral magnitude ^b^	81.1 (60.3–91.4)	90.1 (64–100)	101.1 (88.8–126.1)	0.002 *	0.166	8.6 (−5.5; 30.1)	0.022 **	16.7 (2.2; 39.7)
Median magnitude ratio ^b^	−7 (−7–−7)	−3.90 (−7–−1.33)	−.54 (−1.02–−0.03)	<0.001 *	0.014 **	2.2 (0; 5.1)	0.001 **	3.3 (1; 6.1)

FMA-UE: Fugl-Meyer Assessment upper extremity; good match: FMA-UE >31, SIS-Hand >61; low match: FMA-UE ≤ 31, SIS-Hand ≤ 61; mismatch: FMA-UE > 31, SIS-Hand ≤ 61; Q1: 1st quartile; Q3: 3rd quartile; SIS-Hand: hand domain of the Stroke Impact Scale; 95% CI: 95% confidence interval. * *p* < 0.05, ** *p* < 0.025 (Bonferroni correction for multiple testing). ^a^ One-way ANOVA; post-hoc: Tukey HSD. ^b^ Kruskal–Wallis H; post-hoc: Mann–Whitney *U* test.
